# Powder Bed Fusion Versus Material Extrusion: A Comparative Case Study on Polyether-Ether-Ketone Cranial Implants

**DOI:** 10.1089/3dp.2021.0300

**Published:** 2023-10-10

**Authors:** Yaan Liu, Nan Yi, Richard Davies, Paul McCutchion, Oana Ghita

**Affiliations:** Engineering, College of Engineering, Mathematics and Physical Sciences, University of Exeter, Exeter, United Kingdom.

**Keywords:** PEEK, additive manufacturing, powder bed fusion, fused filament fabrication, cranioplasty

## Abstract

As the choice of additive manufacturing (AM) technologies is becoming wider with reliable processes and a wider range of materials, the selection of the right technology to fabricate a certain product is becoming increasingly difficult from a technical and cost perspective. In this study polyether-ether-ketone cranial implants were manufactured by two AM techniques: powder bed fusion (PBF) and fused filament fabrication (FFF) and their dimensional accuracy, compression performance, and drop tower impact behavior were evaluated and compared. The results showed that both types of specimens differed from the original computer-aided design; although the origin of the deviation was different, the PBF samples were slightly inaccurate owing to the printing process where the accuracy of the FFF samples was influenced by postprocessing and removal of the scaffolds. The cranial implants fabricated using the FFF method absorbed more energy during the compression and impact tests in comparison with the PBF process. The failure mechanisms revealed that FFF samples have a higher ability to deform and a more consistent failure mechanisms, with the damage localized around the puncture head region. The brittle nature of the PBF samples, a feature observed with other polymers as well, led to complete failure of the cranial implants into several pieces.

## Introduction

Polyether-ether-ketone (PEEK) is an engineering thermoplastic with excellent mechanical and thermal performance and high chemical resistance.^1–3^ PEEK also shows good biocompatibility, and is a promising material for biomedical applications such as cranial and spinal implants.^1,4,5^ The radio transparency and the light weight of PEEK makes it a good alternative material to metal implants.^6^

Additive manufacturing (AM) of PEEK offers flexibility for making implant parts with complicated and customized designs. Powder bed fusion (PBF) and material extrusion processes have both been used to produce 3D printed PEEK parts.^4,7,8^ However, the decision on which AM technology to use is not always clear. The choice of AM process is defined by the design requirements, the application, and costs. For example, for applications such as hip and knee implants, there is a need for both dense and porous surfaces and structures.^9^

Dense PEEK structures are required for regions subjected to wear and porous surfaces for regions where bone growth is critical. It could be argued that both AM processes could be used in the manufacture of these parts in different applications. However, in addition to the density of parts, the mechanical performance is also a factor influencing the decision, as the powder bed process normally leads to lower elongation and more brittle structures in comparison with the material extrusion processes.^7,10^ In other applications such as cranial implants, it is less clear which technique would be most appropriate, with two of the important factors being mechanical performance of the printed structure and dimensional accuracy.

PBF was the first AM process used for fabricating PEEK parts. EI Halabi *et al.*^11^ compared the mechanical performance of PEEK cranial implants fabricated through PBF with two different porosity integrated geometries and analyzed the designs by numerical simulation. The implants have shown brittle failure modes with very linear responses. However, this work focused more on the modeling results rather than the process optimizing.

Berretta *et al.*^4^ investigated the effect of four build orientations (horizontal, inverted horizontal, vertical, and oblique 45°) on the geometrical accuracy and mechanical performance of mesh-type PEEK cranial implants. The implants manufactured in the horizontal and inverted horizontal orientations showed the best compressive properties. Compared with the implants built in vertical and oblique 45° orientations, the implants manufactured in horizontal and inverted horizontal orientations showed a tougher failure retaining their integrity outside the region of the piston head. In comparison, the implants fabricated in vertical and oblique orientations had a more brittle failure with a higher number of segments and fragments. Inverted horizontal orientation showed a higher first failure load, maximum load, and total absorbed energy than horizontal orientation. Therefore, all the PBF-fabricated implants in this study were fabricated in inverted horizontal orientation.

The AM techniques vary in equipment and materials costs. The PBF process is costly, whereas material extrusion of polymers, commonly known as fused filament fabrication (FFF), has become a fast-growing AM technique for PEEK fabrication with a significantly lower entry cost. Sharma *et al.*^12,13^ manufactured cranial implants using a FFF printer and their preliminary results showed acceptable dimensional accuracy for craniofacial reconstructions and short processing times (<24 h). Sharma *et al.*^14^ have also tested 10 FFF patient-specific cranial implants under compression forces. The implants showed a semi-brittle/brittle type of failure and variable peak load values, suggesting a more stable printing process is required.

Zhao *et al.*^15^ have utilized FFF to fabricate PEEK cranial implants and the authors compared cranial implant annealed at various temperature with nonheat-treated samples. Zhao *et al.* expected an improvement in mechanical performance as the result of the heat treatment. The results confirmed an improvement of 14% in max load but created also a more brittle failure behavior. All the FFF printed cranial implants showed higher loading-bearing capacity than the parietal cranial bone.

In this study, a comparison in the performance of PEEK cranial implants manufactured by PBF and FFF has been carried out. Most previous studies on cranial implants have tested in quasi-static compression using an indenter loading. However, a skull is less likely to endure a continuous loading and impact loading might be the most realistic experiment. Therefore, the geometrical accuracy, compression properties, and impact behavior of PBF and FFF-fabricated implants were examined by comparison in this study. The results provide guidance for the AM of PEEK implants and future clinical implementation.

## Experimental

### Materials

The same material grade was used for both processes: PEEK 450PF supplied by Victrex Plc, United Kingdom. The powder for PBF had an average particle size of 50 μm. The melting temperature of the PEEK material is 343°C and the glass transition temperature is 143°C. Heat treatment was applied for 24 h at 250°C in an air-ventilated oven to improve the particle flow. More details are given in the previous study.^8^ The powder was then cooled down naturally, to room temperature, and then sieved. The powder was left resting for a day to avoid electrostatic charging before the printing process.

PEEK 450 filament (Victrex Plc, UK) with a diameter of 1.75 mm was used for the FFF printing process.

### Manufacturing methods

**FIG. 1.**
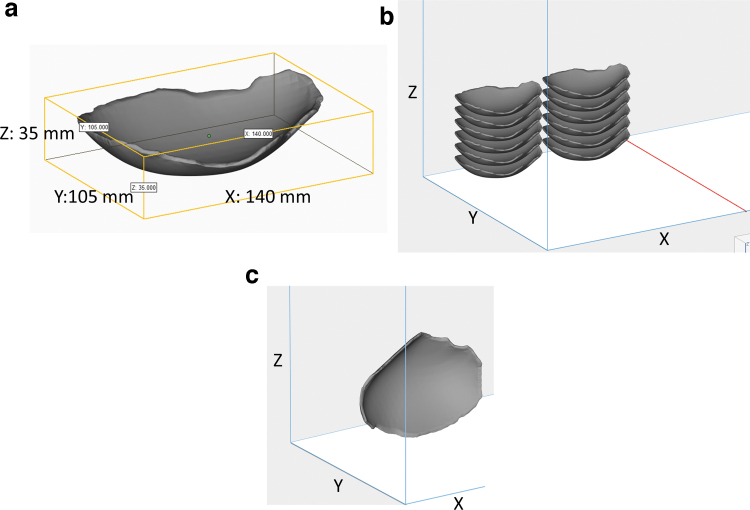
**(a)** CAD model of the cranial implant was provided by Kumovis GmbH; **(b)** PBF build of 10 cranial implants fabricated in inverted horizontal orientation and **(c)** FFF build of cranial implant fabricated in vertical orientation. CAD, computer-aided design; FFF, fused filament fabrication; PBF, powder bed fusion.

The computer-aided design (CAD) model (STL file) of the cranial implant was provided by Kumovis GmbH (Fig. 1a).

Ten cranial implants manufactured by PBF were fabricated in inverted horizontal orientation (Fig. 1b) within the EOS P800 system (EOS, Germany) in reduced chamber configuration mode with a laser power of 9.35 W and laser speed of 1000 mm/s for contour and a laser power of 16.5 W and laser speed of 2550 mm/s for hatching. The hatching distance was 0.2 mm and layer thickness was 0.12 mm. The processing temperature, bed temperature, and sidewalls temperature were 332°C, 310°C, and 315°C, respectively. This orientation was selected based on the results of the previous study carried out by the authors,^4^ which showed that inverted horizontal orientation is the most accurate and strongest in compression tests. A full investigation of the PEEK 450PF powders and its printability for laser sintering has been carried out elsewhere.^8^

Ten cranial implants were manufactured by FFF, using a Kumovis R1 3D printer (Kumovis GmbH, Germany) with a nozzle diameter of 0.6 mm in a vertical orientation (Fig. 1c) to minimize the support materials and postprocessing procedure and provide a smoother surface finish.^13^ The build chamber temperature, build plate temperature, and nozzle temperature were 220°C, 270°C, and 440°C, respectively. The strut width was adaptively adjusted from 0.4 to 0.6 mm with a layer height of 0.35 mm. The printing speed was adaptively adjusted from 1000 to 4000 mm/min. After printing, all the cranial implants were manually cut and ground to remove the support materials and smooth the surface edge surfaces.

The feedstock filaments and powders used for printing were crystalline and all implants were crystalline at the end of the printing processes, no further heat treatment was carried out on any of the samples, PBF or FFF.

### Dimensional accuracy

The dimensional accuracy of the cranial implants was analyzed using a Renishaw Cyclone scanning and measuring system with a 2 mm diameter stainless steel cylindrical probe and an accuracy of 5 μm. Both internal and external surfaces were scanned and compared with the original data of the CAD model using the software package GeomagicStudio v10. The scanned files were manually aligned to the CAD model using the best fit alignment tool and the color deviation maps were obtained with the 3D compare tool. The deviation measurement is a function of GeomagicStudio software and represents the difference between the original STL model and scanned digital mesh.

### Quasi-static compression test

**FIG. 2.**
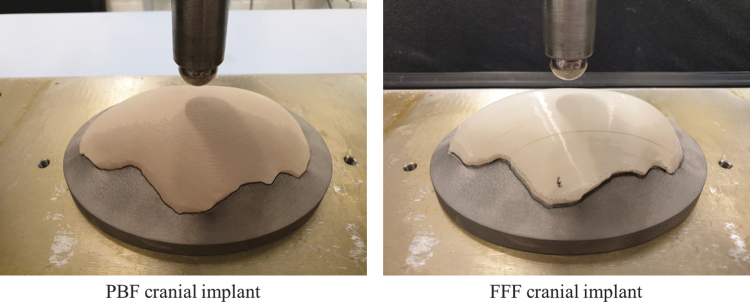
Compression testing for PBF and FFF cranial implants. The cranial implants were placed on a metal custom-made sample holder.

The PBF and FFF-fabricated cranial implants were tested in static uniaxial compression loading using Shimadzu AGS-20 kNX with a 20 kN load cell, test speed of 1 mm/min, and a hemispheric indenter with a diameter of 10 mm. The specimens were placed on a custom-made laser sintered metallic sample holder (Fig. 2). The test started with a 5 mm distance between the indenter and the PBF specimen without any contact and then the force was applied on the center top of the specimen until structural failure. For the FFF specimens, the indenter started from the same onset position to compare the dimension variation between the PBF and FFF specimens. Five repeats were performed for both the PBF and FFF-fabricated specimens. The implants were tested until the indenter completely penetrated the specimens.

### Drop tower impact test

The drop tower tests were performed on an Instron CEAST 9350 drop tower. A hemispheric nose impactor with a diameter of 20 mm as an instrumented striker, a load cell of 4.5 kN, and a weight of 2.45 kg were used. The specimens were placed on the same metallic custom-made sample holder as the one used for quasi-static compression testing. Experiments were conducted at the same height of 0.2 m and impact energy of 15 J. Five repeats were performed for both the PBF and FFF-fabricated specimens.

### Scanning electron microscopy

Scanning electron microscopy (SEM) images of the fractured structures were acquired by a Tescan VEGA3 SEM (Tescan, United Kingdom). Both printed surfaces and the cross-section of fractured parts were characterized. The fractured parts were pasted on conductive carbon tape and then sputter coated with 15 nm of Cr to reduce surface charging. The secondary electron imaging was carried out using an accelerating voltage of 20 kV.

## Results and Discussion

### Dimensional accuracy

**FIG. 3.**
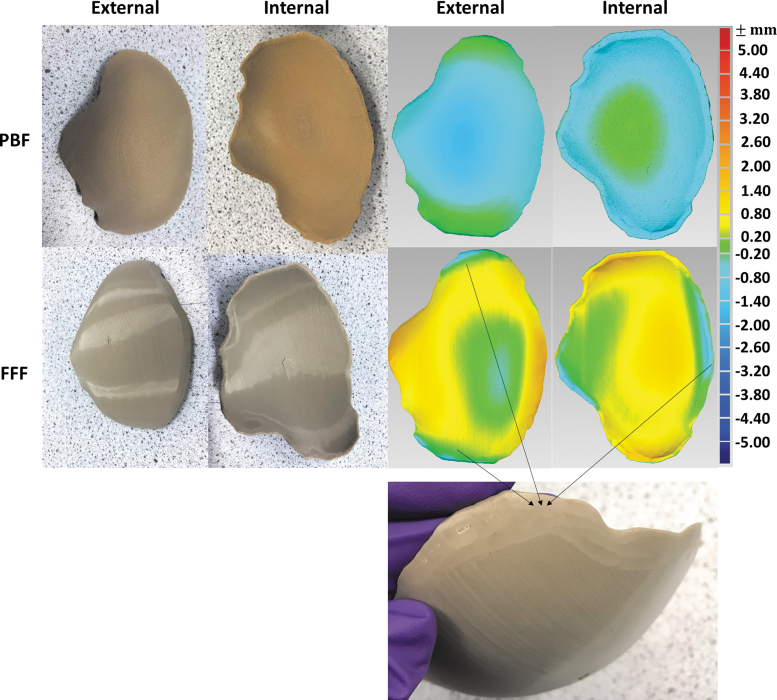
The printed PBF and FFF cranial implant specimens and the color deviation maps of the dimensional accuracy for the external and internal surfaces of PBF and FFF cranial implants. The *red* regions represent positive deviations and the *blue* regions show negative deviations. The negative deviations on the edge regions of FFF specimen were owing to the manual cutting and grinding to remove support materials.

The printed specimens and color deviation maps of the dimensional accuracy measured for PBF and FFF-fabricated cranial implants are given in Figure 3. The blue regions represent negative deviations and the red regions show positive deviations.

For the PBF specimen, both external and internal surfaces printed in the inverted horizontal show negative deviations (blue) relating to the shrinkage. No shrinkage factors were applied in the PBF printing process. The internal surface revealed a maximum difference of −0.80 mm. The external surface showed a higher maximum difference, which is −1.40 mm. These difference values are higher than previous work of Berretta *et al.*^4^ (± 0.40 mm at most for the PBF mesh-type PEEK cranial implant in the inverted horizontal orientation), and future investigations could aim to increase accuracy by optimizing the shrinkage factors used during the PBF process.

The FFF specimens generally show positive deviations of dimensional accuracy. Most areas show a difference within 0.80 mm. It was noticed that the edge regions for both internal and external surfaces show negative deviations (−0.80 mm) owing to the manual cutting and grinding to remove the support materials and smoothen the edge surfaces. The dimensional differences recorded in the Kumovis prints (−0.80 to +0.80 mm) are higher than the FFF-printed cranial implants of Sharma *et al.*,^12^ which had dimensional differences of −0.30 to 0.22 mm. The negative deviations on the edge regions affected the sample fitting on the metallic holder for the mechanical tests.

### Quasi-static compression test

**FIG. 4.**
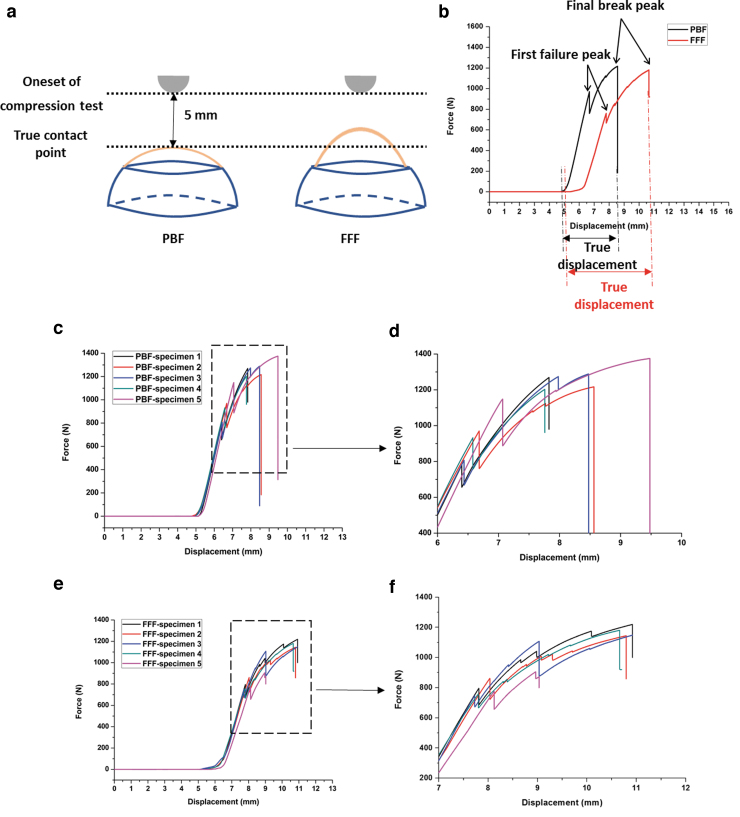
**(a)** Illustration of the experimental set-up of quasi-static compression test for PBF and FFF cranial implant specimens; **(b)** illustration of first failure peak and final break peak in the load–displacement curve; true displacement was defined as the displacement from the displacement at 1 N load force to the displacement at the final break; **(c)** five repeat load–displacement curves of PBF specimens and **(d)** zoomed in curves; and **(e)** five repeat load–displacement curves of FFF cranial implants and **(f)** zoomed in curves.

The set-up of the quasi-static compression test is given in Figure 4a. The specimens were placed on a custom-made laser sintered metallic sample holder (Fig. 2). The test began with a 5 mm distance between the indenter and the PBF specimen without any contact. The indenter started from the same onset position for the FFF specimens. It was noticed that the PBF specimens had a better fit in the metallic holder than the FFF specimens owing to the manual cutting of the FFF samples. Therefore, the contact points of the PBF and FFF samples were different (given in Fig. 4a).

Figure 4b provides the typical force–displacement curves of the PBF and FFF specimens. The printed cranial implants were tested under uniaxial loading until the cranial failure. There are three stages for each curve. Stage 1 is an initialization phase showing the indenter travel from the onset point to the true contact point; Stage 2 is a preloading phase showing the implant specimen was pushed by the indenter against the sample holder, and Stage 3 is a compression phase showing the compression behavior.

For both PBF and FFF cranial implants, the curves show first failure peaks and final break peaks. The first failure peak was identified as the first small peak in the load–displacement curve. The first failure peak is created by the breakage of the external layer on the nonimpacted side of the impactor. The cracks then propagate throughout the entire implant and break off from the specimen creating the final break peak.

**FIG. 5.**
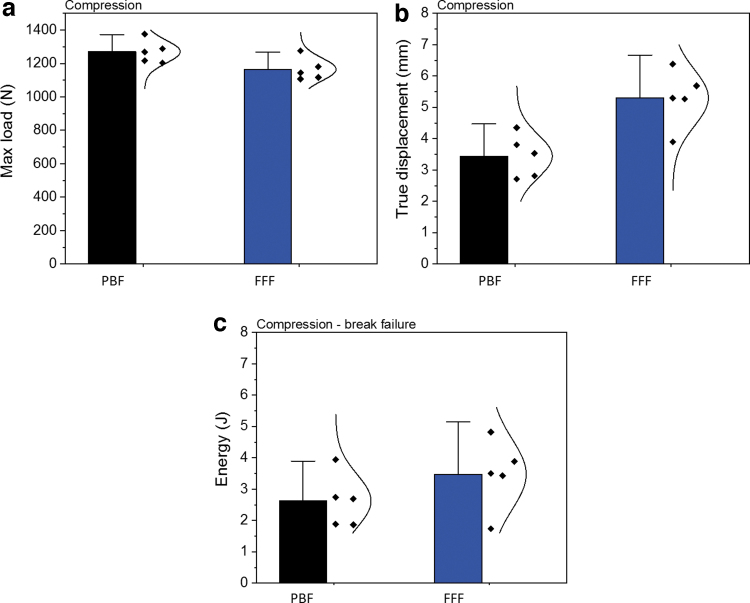
**(a)** Maximum load, **(b)** true displacement, and **(c)** total energy of the PBF and FFF cranial specimens from the quasi-static compression test.

To compare the displacements between the PBF and FFF specimens, the true displacement is defined as the displacement starting from the displacement at 1 N load force to the displacement at the final break (illustrated in Fig. 5b). Five repeats were performed for both PBF and FFF cranial specimens, and the load–displacement curves are given in Figure 4c and e. Figure 4d and f provides the zoomed-in images. In addition to the first failure peak and the final break peak, the FFF specimens seem to go through a number of cracks, whereas PBF specimens perform at most a two-step failure mechanism, associated with a more sudden break and more brittle failure pattern.

In Figure 5a, maximum load is defined as the highest load applied on the specimens during the test. For both types of specimens, the maximum load values are much higher than the parietal cranial bone data (793.7 N) experimentally measured by Motherway *et al.*^16^ at 1 mm/s. The true displacement values of both types of specimens are given in Figure 5b. The results of the total energy absorbed by the implants including the ultimate failure are given in Figure 5c. The PBF specimens can bear a higher load before the final break compared with the FFF specimens but had a lower true displacement and lower total energy absorbed.

**FIG. 6.**
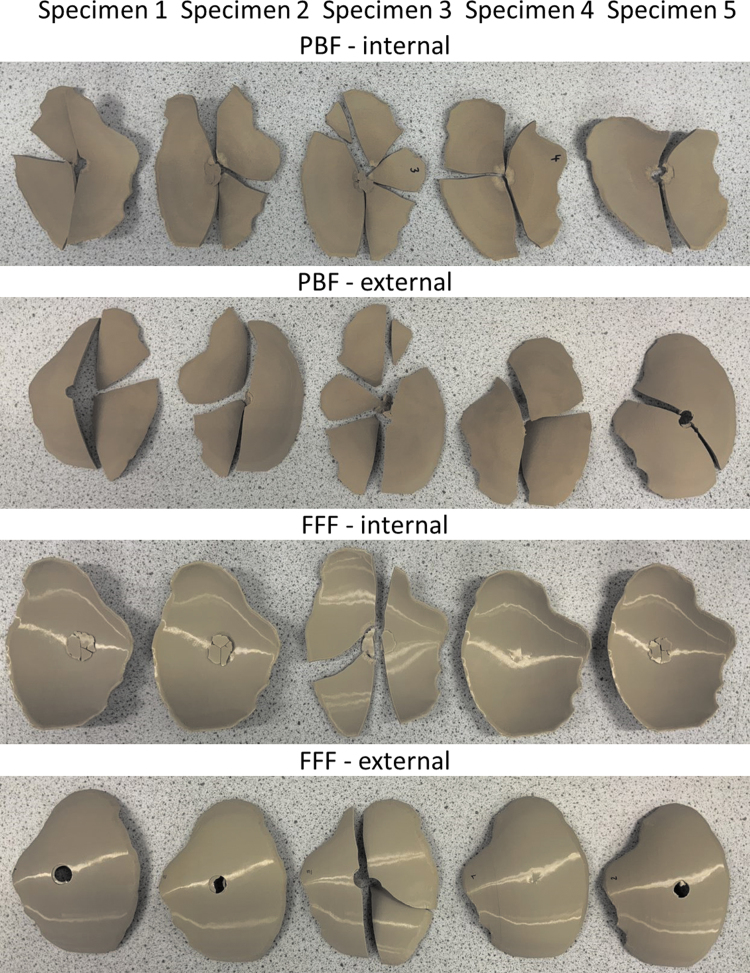
Fractured PBF and FFF cranial implant specimens after compression tests. PBF cranial implants shattered into several segments and fragments, whereas four of FFF cranial implants retained their integrity except for the regions of penetration of the indenter suggesting a tougher failure than the PBF specimens.

Figure 6 provides the fractured implants of PBF and FFF specimens. The PBF cranial implants shattered into several fragments, suggesting a brittle failure, whereas four of the five FFF cranial implants retained their integrity except for the regions of penetration of the indenter, indicating a tougher failure than the PBF specimens. A brittle failure would be a potential risk for a medical application as it may cause damage to surrounding tissue and the cranial bone.

**FIG. 7.**
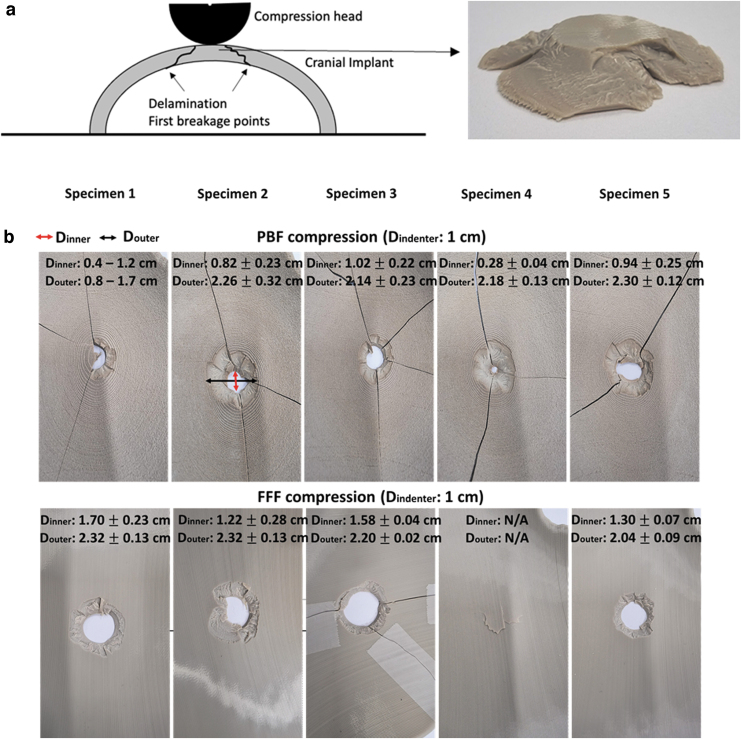
**(a)** Illustration of the first failure in the FFF samples tested in compression may be caused by the breakage of the external layer on the nonimpacted side of the impactor or delamination. The cracks then propagate in a conical shape from the nonimpacted side toward the impacted side in contact with the compression head; and **(b)** presents the re-constructed broken specimens (PBF above and FFF below) after the compression tests together with the inner and outer diameters of the indentations.

The first failure peaks noticed in the FFF samples tested in compression may be caused by the breakage of the external layer on the nonimpacted side of the impactor or delamination. Following this delamination and breakage points, the cracks propagate in a conical shape advancing at an angle from the nonimpacted side toward the impacted side in contact with the compression head. Figure 7a provides the initial breakage points. The broken specimens were reconstructed and is given in Figure 7b, where the conical delamination and breakage mechanism is visible in all compression-tested FFF specimens. Similar features and mechanism of failure were noticed in other composite structures tested in compression.^17–19^ Here, the effect is even more pronounced owing to the concave shape of the specimen.

In contrast, the compression-tested PBF specimens, the conical delamination was noticed but the specimens cracked throughout before full penetration of the compression head, showing a much more brittle structure. The comparison in the inner diameter of the indentation created by the compression test (Fig. 7b) shows clearly that the PBF cranial implants crack prematurely across the entire structure.

A summary of the literature for the quasi-static compression testing of cranial implants is given in Supplementary Table S1. In some cases, the dimensions vary significantly, therefore the results are difficult to compare. The results obtained in this work showed higher max load and displacement than parietal cranial bone values presented in the literature,^16^ but the values were lower than Zhao *et al.*'s work on FFF cranial implants.^15^ The differences may be owing to the different building orientations. Samples of Zhao *et al.* were built in a horizontal orientation, whereas in this study the FFF samples were printed vertically. The thickness of cranial implants could be another factor in the differences noticed, although the thickness dimensions in the work of Zhao *et al.* are not presented.

Sharma *et al.*^14^ have also measured the compression properties of 10 FFF patient-specific cranial implants. The implants were printed in vertical orientation and showed more brittle types of failure with a mean peak load of 798 ± 211 N and displacement of 2.54 ± 0.56 mm, which could be owing to the different PEEK material (Evonik PEEK filament) and FFF printer (Apium M220) used.

In the case of PBF implants, the PBF samples fabricated in this study showed better compression performance than previous PBF-fabricated PEEK implants possibly owing to the solid structures of the cranial implants used here rather than the mesh implants tested in previous studies.^4,11^ Lethaus *et al.*^20^ reported higher compression results for the PEEK cranial implant but the manufacturing method is unknown.

### Drop tower impact test

The drop tower impact test was conducted at the same height of 0.2 m, with an impact energy of 15 J to ensure all cranial implants (five PBF and five FFF implants) were fractured.^21^ Owing to the size variations of the PBF and FFF specimens, the true contact positions were different (given in Fig. 4a), resulting in variations in the onset impact velocity for the two types of specimens (Table 1). A higher variation in impact velocity for the FFF samples is because of the dimensional variance caused by manual cutting and removing of support materials.

**Table 1.** Impact Energy Applied on the Drop Tower Impact Test and the Corresponding Impact Velocity on Powder Bed Fusion and Fused Filament Fabrication Cranial Implant Specimens

	Impact energy applied (J)	Impact velocity (m/s)
PBF	15	2.18 ± 0.08
FFF	15	1.91 ± 0.17

FFF, fused filament fabrication; PBF, powder bed fusion.

**FIG. 8.**
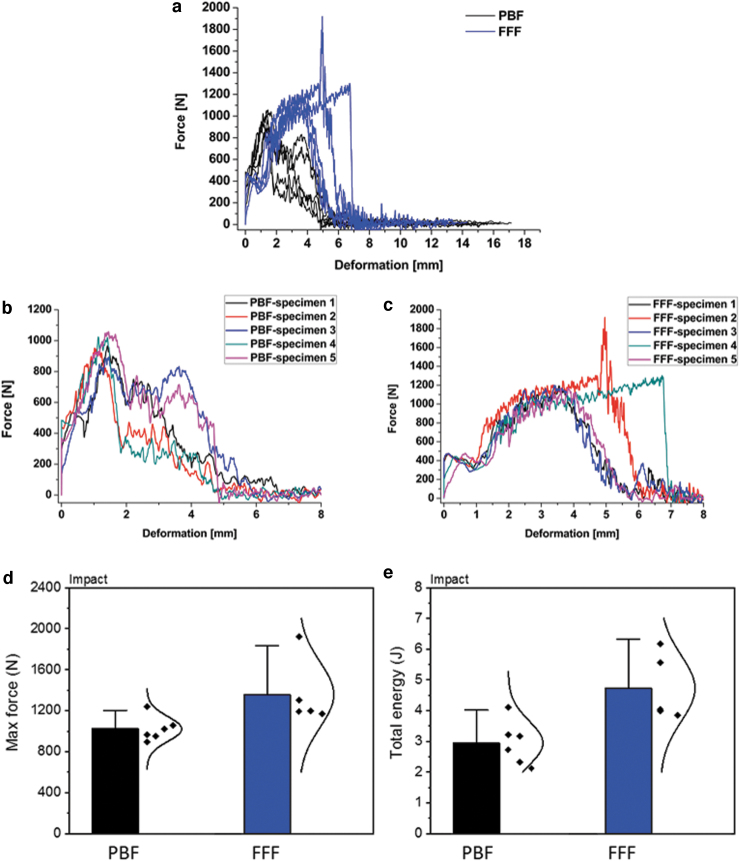
**(a)** Force versus deformation curves for PBF and FFF cranial implants from the drop tower impact test; **(b)** zoomed in curves of PBF specimens; **(c)** zoomed in curves of FFF specimens; **(d)** maximum force and **(e)** total energy absorbed for the impact tests.

The force–deformation curves for all specimens from the impact tests are given in Figure 8a. For better clarity, the region of high impact was magnified and is given in Figure 8b and c. Overall, the PBF specimens show maximum forces at ∼1 mm with two of the PBF specimens showing an additional force peak at ∼4 mm. Unlike PBF, all FFF specimens show a small and repeatable force peak at low deformations at 0.5–0.8 mm, followed by maximum forces at higher deformations at ∼4 mm. In the case of the compression-tested FFF specimens, the fracture peak was less steep and wider, with the structure deforming significantly before the penetration of the compression head through the implant.

Figure 8d and e show the max force and total energy (the area under the force–deformation curves) for the PBF and FFF cranial specimens after the drop tower impact test. FFF cranial specimens show higher max force and total energy than the PBF specimens, which is again consistent in behavior with the compression tests.

**FIG. 9.**
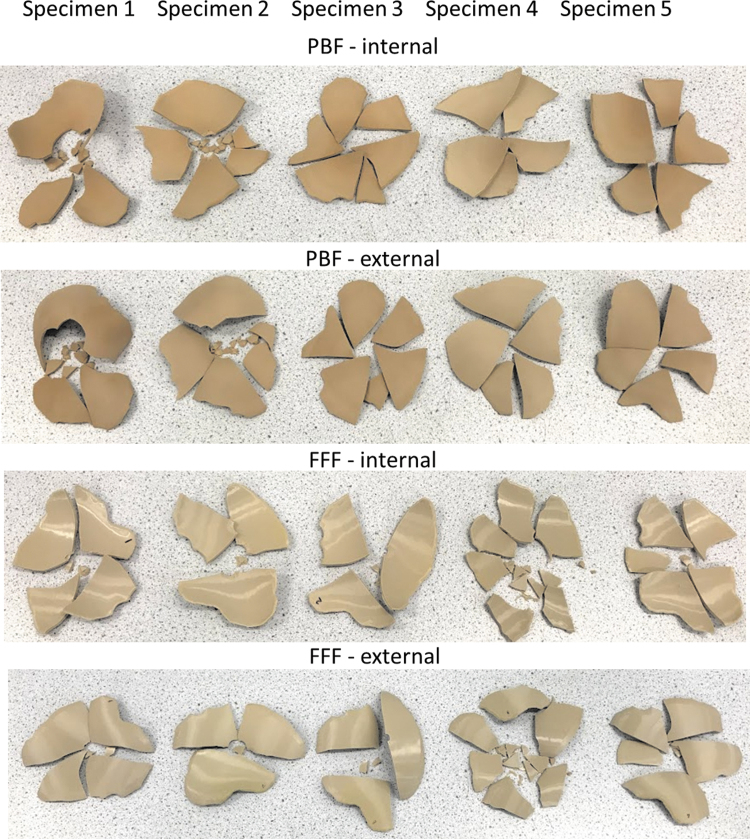
Fractured PBF and FFF cranial implant specimens after drop tower impact tests. PBF and FFF cranial implants shattered into several segments and fragments.

Figure 9 shows the fractured implants of PBF and FFF specimens after the drop tower impact tests. All the PBF and FFF cranial implants fractured and shattered into several fragments.

There are only a few experimental studies that have tested cranial implants under impact loading and these are not on PEEK but on hydroxyapatite cement cranial implants,^22^ titanium,^23^ and calcium phosphate–titanium^24^ materials. To the authors' knowledge, this is the first study presenting results on printed PEEK cranial implants under this type of loading.

Ambrogio *et al.*^23^ manufactured titanium cranial implants by superplastic forming and single-point incremental forming. The implants did not fracture during the impacting tests under impact energies of 4.5 and 13.5 J. The peak loads ranged from 1.4 to 4.7 kN under different impact energy and with different implant thickness and Ti alloys. Lewin *et al.*^24^ fabricated calcium phosphate–titanium implants based on printed titanium embedded in self-setting calcium phosphate. The maximum impact loads were ∼0.8 kN under impact energy of 5.75 J. Supplementary Table S2 summarizes the literature for the impact testing on cranial implants.

**FIG. 10.**
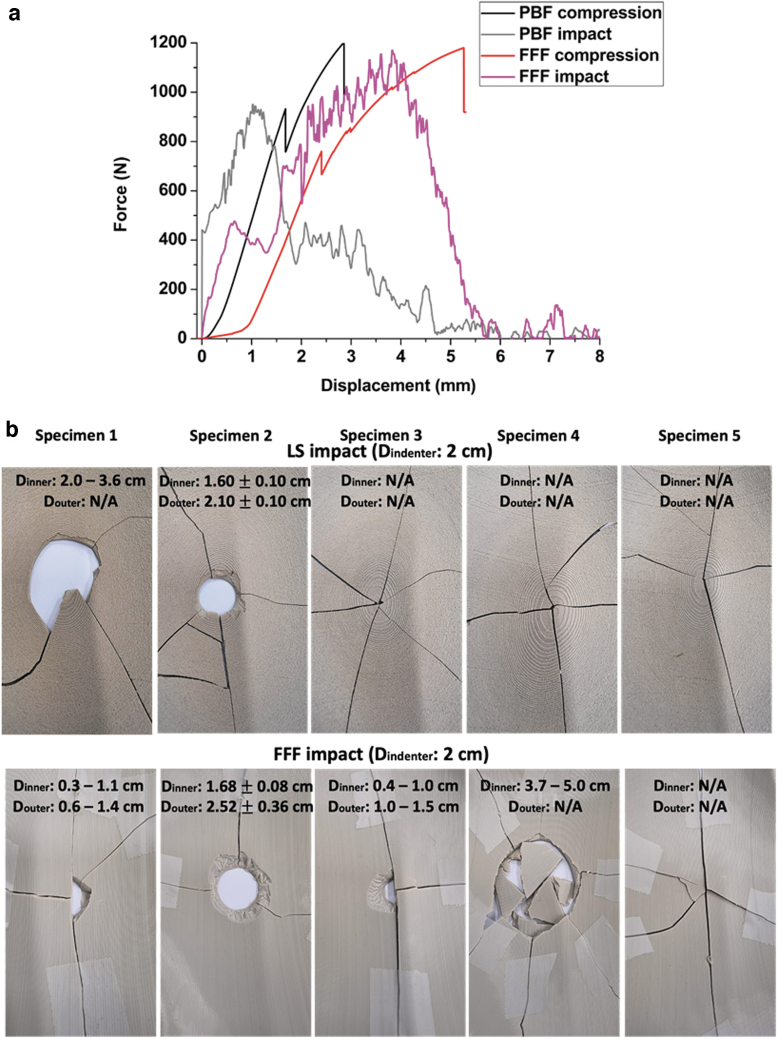
**(a)** A comparison of force–displacement curves between quasi-static compression test and drop tower test on PBF and FFF cranial implant specimens; and **(b)** the reconstructed broken specimens (PBF above, FFF below) after the drop tower impact tests and the inner and outer diameters of the indentations.

In an attempt to better understand the failure mechanisms of the two types of cranial implants, the force–displacement curves of the two tests (the quasi-static compression test and drop tower impact test) were plotted together in Figure 10a. The impact velocity differs depending on the impact methods, which are 0.016 m/s for the quasi-static compression test and ∼2 m/s for the drop tower impact test (Table 2). The effect of impact velocity is visible in Figure 10a, the final break point is shifted to higher displacements (by ∼2 mm) in the case of the compression test. The slow impact velocity of the quasi-static compression test (0.016 m/s) creates a longer crack path and delays the final break of both types of specimens.

**Table 2.** A Comparison Between Quasi-Static Compression Test and Drop Tower Test on Powder Bed Fusion and Fused Filament Fabrication Cranial Implant Specimens

	PBF compression	PBF impact	FFF compression	FFF impact
Impact velocity (mm/s)	0.016	2.18	0.016	1.91
Max force (N)	1271 ± 69	978 ± 64	1369 ± 130	1356 ± 319
Total energy (J)	2.62 ± 0.85	2.71 ± 0.49	3.47 ± 1.12	3.66 ± 1.07

In the case of the drop tower test, the high impact velocity (2 m/s) leads to full sample failure across the entire structure with less repeatability in the failure patterns (Fig. 10b). Table 2 summarizes the max forces and total energy obtained from both testing methods. It was noticed that the total energy remained similar regardless of the static or dynamic impact tests. Similar results have been found in other literature.^17–19^

### SEM images

**FIG. 11.**
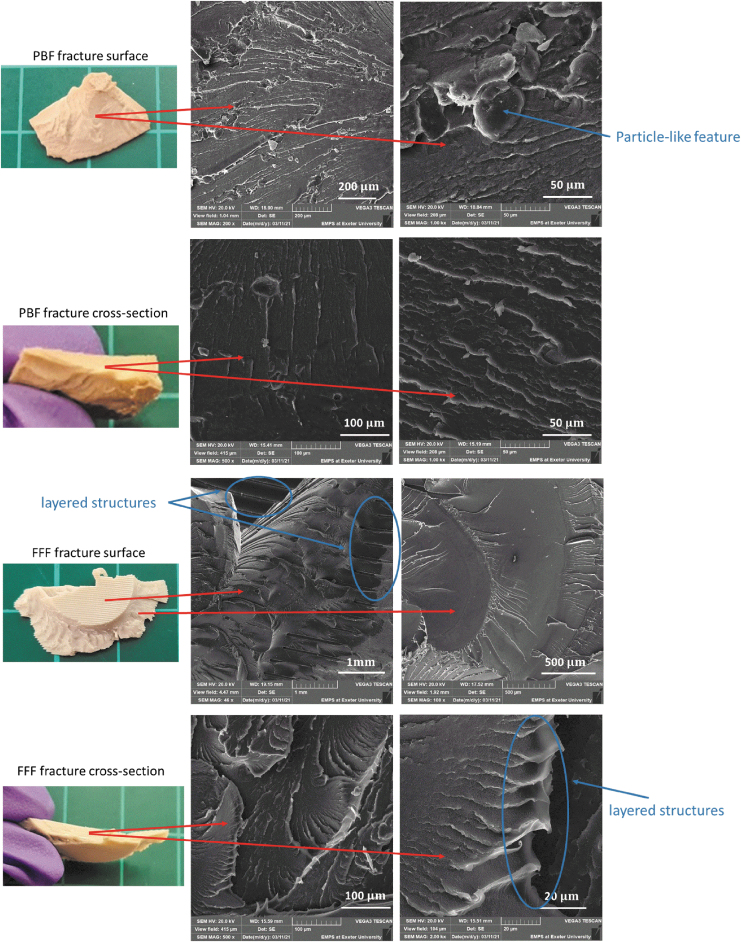
SEM images of fractured structures PBF and FFF specimens after the quasi-static compression tests. Brittle fracture surfaces were observed on the PBF specimen. Printing layers were observed on FFF specimen, and more ductile fracture behavior was found from the cross-section area. SEM, scanning electron microscopy.

Figure 11 provides the SEM images of the fracture surfaces for the PBF and FFF cranial implant specimens obtained from the quasi-static compression tests. The top surface and the cross-section surface of the fractured parts were observed, with PBF fracture surfaces indicating a more brittle failure than FFF, and the presence of particle-like features were found, which may be an indication of partially molten PEEK particles. The SEM images of the FFF fracture surfaces do show a layered structure in the printed layer direction, but no visible delamination phenomenon was overserved during the mechanical tests for either PBF or FFF specimens, which indicates that layer-to-layer bonding in both processes was good.

## Conclusions

A comparison in the performance of PEEK cranial implants manufactured by PBF and FFF were carried out. The dimensional accuracy, compression performance, and impact behavior were examined.

The dimensional accuracy of both types of samples needs improvement although the causes are slightly different. In the case of PBF-fabricated implants, the dimensional accuracy has been affected by the printing process. The dimensional accuracy of FFF specimens was affected by the postprocessing such as removal of support materials.

The mechanical performance exhibited in the compression and impact tests suggest that FFF implant specimens, manufactured using the processes followed in this investigation, can sustain higher forces and overall energy, and deform significantly more than the PBF equivalent while exhibiting a more ductile fracture mechanism. It has been found that the total energy was not significantly affected by testing methods (quasi-static compression or drop tower impact tests) but the failure mechanisms were different when comparing the tests and printed specimens.

## Supplementary Material

Supplemental data

Supplemental data
