# Supplementary material for: Powder Bed Fusion Versus Material Extrusion: A Comparative Case Study on Polyether-Ether-Ketone Cranial Implants
Source: 3D Print Addit Manuf. 2023 Oct 10;10(5):941–54. doi: 10.1089/3dp.2021.0300 (PMC10599438; doi:10.1089/3dp.2021.0300)
Supplement: Supplemental data [file Supp_TableS1.docx]

**Table S1.** Summary of quasi-static compression tests on cranial implants.

| **Reference** | **Materials and methods** | **Specimen details** | **Test conditions** | **Max load** | **Max displacement** | **Total energy** | **Failure behaviour** |
| --- | --- | --- | --- | --- | --- | --- | --- |
| Motherway et al.^16^ | Parietal cranial bone | **Sample size:** 6 $\times$ 1 cm  Adult cranial bones.  Various thickness from 4.4 to 8.6 mm. | **Quasi-static bending:**  1mm/min | **Right parietal:**  793.7 N  **Left parietal:**  584.3 N  **Frontal:**  1035.7 N | Less than 1 mm | N/A | N/A |
| This work | PEEK via PBF and FFF | Cranial implant model provided by Kumovis GmbH. The thickness of the model varies roughly from 2.3 to 4.5 mm. | **Quasi-static**:  1 mm/min  **Indenter:**  hemispherical, $\emptyset$ = 10 mm  **Support:** metal | **PBF**:  1270.7 $\pm$ 68.5  **FFF:**  1164.9 $\pm$ 68.0 | **PBF:**  3.44 $\pm$ 0.69  **FFF:**  5.31 $\pm$ 0.91 | **PBF:**  2.62 $\pm$ 0.85  **FFF:**  3.47 $\pm$ 1.12 | **PBF:**  several fragments  **FFF:**  kept integrity |
| Zhao et al.^15^ | PEEK via FFF built in horizontal orientation with heat treatment | 3D skulls reconstructed by Mimics software. Thickness unknown. | **Quasi-static**:  1 mm/min  **Indenter:** hemispherical  $\emptyset$ = 10 mm  **Support:** flat anvil | **Without heat treatment:**  7000 N  **With heat treatment:**  8000 N | **Without heat treatment:**  18 mm  **With heat treatment:**  8 mm. | N/A | **Without heat treatment:**  kept integrity  **With heat** **treatment:**  several fragments |
| Sharma et al.^12^ | PEEK via FFF built in vertical orientation | Patient-specific cranial model. Typical thickness from 3-5 mm. | **Quasi-static**:  1 mm/min  **Indenter:**  hemispherical, $\emptyset$ = 10 mm  **Support:** polymer | 798 $\pm$ 211 N | 2.54 $\pm$ 0.56 mm | N/A | Kept integrity with layer delaminating |
| Berretta et al.^4^ | Mesh PEEK implant via PBF built in four orientations | Patient cranial model with meshed structure. Thickness unknown. | **Quasi-static**:  1 mm/min  **Indenter:**  Hemispherical, $\emptyset$ = 10 mm  **Support:** polymer | **Horizontal:**  620 N  **Vertical:**  320 N  **Oblique:**  600 N  **Inverted horizontal:**  790N | N/A | **Horizontal:**  1.8 J  **Vertical:**  1.1 J  **Oblique:**  2.1 J  **Inverted horizontal:**  2.4J | **Horizontal:**  kept integrity  **Vertical:**  several fragments **Oblique:**  several fragments **Inverted horizontal:**  kept integrity |
| El Halabi et al.^11^ | Mesh PEEK implant via PBF | Two designs of meshed cranial implants. Thickness unknown. | **Quasi-static:**  0.08 mm/min  **Indenter:** hemispherical  $\emptyset$ = 3 mm  **Support:**  Horizontal testing grip | **Implant 1:**  608 $\pm$44 N  **Implant 2:** 1028 $\pm$ 69 N | **Implant 1:**  1.77 $\pm$ 0.12 mm  **Implant 2:**  1.69 $\pm$ 0.13 mm | N/A | N/A |
| Lethaus et al.^20^ | Ti-6Al-4V and PEEK Optima, customised solid | Cranial implant with an area of 100 cm2 and a wall thickness of 6 mm. | **Quasi-static:**  1.925 mm/min  **Indenter:** flat  $\emptyset$ = 50 mm  **Support:**  polyamide skull model | **Ti-6Al-4V:**  50 kN  **PEEK:**  24 kN | **Ti-6Al-4V:**  5.7 mm  **PEEK:**  8 mm | N/A | **Ti-6Al-4V:** no deformation but damage to fixation screws  **PEEK:** several fragments |
| Lewin et al.^24^ | Calcium phosphate- Ti-6Al-4V composite | Two designs of meshed cranial implants. The titanium structures were additively manufactured by PBF and embedded in Calcium phosphate materials. | **Quasi-static:**  1 mm/min  **Indenter:** flat  $\emptyset$ = 40 mm  **Support:**  metal  Silicone rubber sheet between indenter and implant | **Design 1:**  808$\pm$ 29 N  **Design 2:**  846 $\pm$ 40 N | **Design 1:**  6.2 $\pm$ 0.7 mm  **Design 2:**  11.7 $\pm$ 0.5 mm  (At peak load, before the force had dropped to 50% of the peak load) | **Design 1:**  7.3 $\pm$ 0.4 J  **Design 2:**  8.4 $\pm$ 0.5 J  (Energy absorbed at 15 mm) | Titanium deformed/cracked |
