# Supplementary material for: Powder Bed Fusion Versus Material Extrusion: A Comparative Case Study on Polyether-Ether-Ketone Cranial Implants
Source: 3D Print Addit Manuf. 2023 Oct 10;10(5):941–54. doi: 10.1089/3dp.2021.0300 (PMC10599438; doi:10.1089/3dp.2021.0300)
Supplement: Supplemental data [file Supp_TableS2.docx]

**Table S2.** Summary of drop tower impact tests on cranial implants,

| **Reference** | **Materials and methods** | **Specimen details** | **Test conditions** | **Max load** | **Total energy** | **Failure behaviour** |
| --- | --- | --- | --- | --- | --- | --- |
| This work | PEEK via PBF and FFF | Cranial implant model provided by Kumovis GmbH. The thickness of the model varies roughly from 2.3 to 4.5 mm. | **Drop tower energy:** 15 J **Indenter:** hemispherical  $\emptyset$ = 20 mm | **PBF**:  1015.07$\pm$ 133.29 N  **FFF:**  1355.82 $\pm$ 319.46 N | **PBF:**  2.90 $\pm$ 0.79 J  **FFF:**  4.72 $\pm$ 1.07 J | Fracture |
| Matic et al.^22^ | HA cement | Fresh cadaver heads were used as the model with 3$\times$3 cm square shape. | **Drop tower energy:** 5.1 to 9.0 J  **Indenter:** hemispherical  $\emptyset$ = 9.8 cm  The specimen (9 cm^2^) was smaller than the indenter. | 587 to 2425 N | N/A | Some fracture |
| Ambrogio et al.^23^ | Ti-6Al-4V via super plastic forming and single-point incremental forming; with different thickness (1 mm and 1.5 mm) and different alloys | Skull models with different thickness of 1 mm and 1.5 mm using different grades of alloys | **Drop tower energy:** 4.5 J and 13.5 J  **Indenter:** hemispherical  $\emptyset$ = 20 mm | 1.4 o 4.7 kN | 2.65 to 1.27 J | No fracture |
| Lewin et al.^24^ | Calcium phosphate- Ti-6Al-4V composite | Two designs of meshed cranial implants. The titanium structures were additively manufactured by PBF and embedded in Calcium phosphate materials. | **Drop tower energy:** 5.75 J  **Indenter:** hemispherical  $\emptyset$ = 40 mm | **Design 1:**  852$\pm$ 34 N  **Design 2:**  814 $\pm$ 4=13 N | **Design 1:**  3.1 $\pm$ 0.8 J  **Design 2:**  4.4 $\pm$ 1.8 J | No titanium fracture |
